# Data-Driven Identification of Early Cancer-Associated Genes via Penalized Trans-Dimensional Hidden Markov Models

**DOI:** 10.3390/biom15020294

**Published:** 2025-02-16

**Authors:** Saeedeh Hajebi Khaniki, Farhad Shokoohi

**Affiliations:** 1 Department of Biostatistics, School of Health, Mashhad University of Medical Sciences, Mashhad 9137673119, Iran; hajebis971@mums.ac.ir; 2 Department of Mathematical Sciences, University of Nevada Las Vegas, Las Vegas, NV 89154, USA

**Keywords:** DNA methylation, accelerated failure time, penalized regression, bisulfite sequencing, survival analysis

## Abstract

Colorectal cancer (CRC) is a significant worldwide health problem due to its high prevalence, mortality rates, and frequent diagnosis at advanced stages. While diagnostic and therapeutic approaches have evolved, the underlying mechanisms driving CRC initiation and progression are not yet fully understood. Early detection is critical for improving patient survival, as initial cancer stages often exhibit epigenetic changes—such as DNA methylation—that regulate gene expression and tumor progression. Identifying DNA methylation patterns and key survival-related genes in CRC could thus enhance diagnostic accuracy and extend patient lifespans. In this study, we apply two of our recently developed methods for identifying differential methylation and analyzing survival using a sparse, finite mixture of accelerated failure time regression models, focusing on key genes and pathways in CRC datasets. Our approach outperforms two other leading methods, yielding robust findings and identifying novel differentially methylated cytosines. We found that CRC patient survival time follows a two-component mixture regression model, where genes *CDH11*, *EPB41L3*, and *DOCK2* are active in the more aggressive form of CRC, whereas *TMEM215*, *PPP1R14A*, *GPR158*, and *NAPSB* are active in the less aggressive form.

## 1. Introduction

Cancer remains a prevalent malignancy with a complex etiology involving genetic mutations, epigenetic alterations, and environmental influences [1]. Among various cancers, colorectal cancer (CRC) is the third most prevalent cancer worldwide [2]. Despite advancements in diagnostic and treatment modalities, the detailed mechanisms underlying CRC initiation and progression remain only partially elucidated [3]. Many CRC cases go undetected until advanced stages, where treatment options become limited [4,5]. Thus, CRC is one of the leading causes of cancer-related death worldwide [2]. Early cancer diagnosis significantly improves patients’ survival time. Since cancer initiation can be driven by epigenetic processes, these early changes can be used to detect cancers in the beginning stages [5]. One of the widely accepted epigenetic mechanisms is ‘DNA methylation’. Aberrant DNA methylation is a hallmark of early-stage carcinogenesis and plays a central role in regulating gene expression, cellular differentiation, and disease progression [6]. More specifically, hypo-methylation in promoter regions may contribute to genomic instability and the activation of oncogenes [7], while the silencing of tumor suppressor genes via hyper-methylation in CpG islands and promoter regions has been introduced as a key mechanism in the progression of many cancers, including CRC [8]. Although some epigenetic changes have been identified as useful biomarkers for early CRC detection [9,10], our understanding of the specific alterations involved in the initial neoplastic progression remains incomplete. Further research is needed to refine and expand the list of epigenetic markers, improving their diagnostic and prognostic potential.

Aberrant Crypt Foci (ACF) are pre-polyp abnormalities, a subset of which may be considered the earliest identifiable precursors to CRC tumors [11]. This occurrence likely represents a fundamental molecular and pathophysiological event in the initiation and development of CRC [12,13], offering a unique opportunity to study the epigenetic alterations associated with the onset of CRC [14]. Some studies have identified methylation alterations in several genes within human ACF, including *MINT1*, *MINT2*, *MINT31*, *CDKN2A*, *RASSF1A*, homeobox genes, and *PRC2* [15,16,17]. However, these results are based on conventional statistical methods such as ANOVA and *t*-tests. Although ANOVA, *t*-tests, and other similar statistical methods can detect differences, they are not designed to assess prognostic value. These methods lack the ability to account for autocorrelation among nearby CpGs and are particularly sensitive to skewed distributions and outliers, both of which are prevalent in DNA methylation datasets. Additionally, these approaches focus solely on mean methylation ratios, disregarding read-depth within the CpGs [18]. Other inherent issues further complicate their use, as extensively documented in the literature [19,20,21].

Powerful statistical models such as hidden Markov models (HMMs) are more suitable for addressing these limitations, including the effective capture of local autocorrelation [18]. Building on HMMs, Shokoohi et al. [21] proposed a penalized trans-dimensional HMM for the identification of differentially methylated cytosine (DMC), implemented in the R-package DMCTHM (Version 0.1). This method represents a significant advancement by introducing a Bayesian framework that simultaneously accommodates the estimation of HMM order and parameters while penalizing over- and underfitting [21]. This approach allows for the detection of methylation changes even in the presence of missing data, which is a common occurrence in sequencing-based methylation datasets. Consequently, DMCTHM is capable of identifying previously unrecognized methylated positions that can enhance our understanding of CRC biological pathways and can form candidate genes playing a significant role in the prognosis of CRC in its early stages.

**Figure 1:**
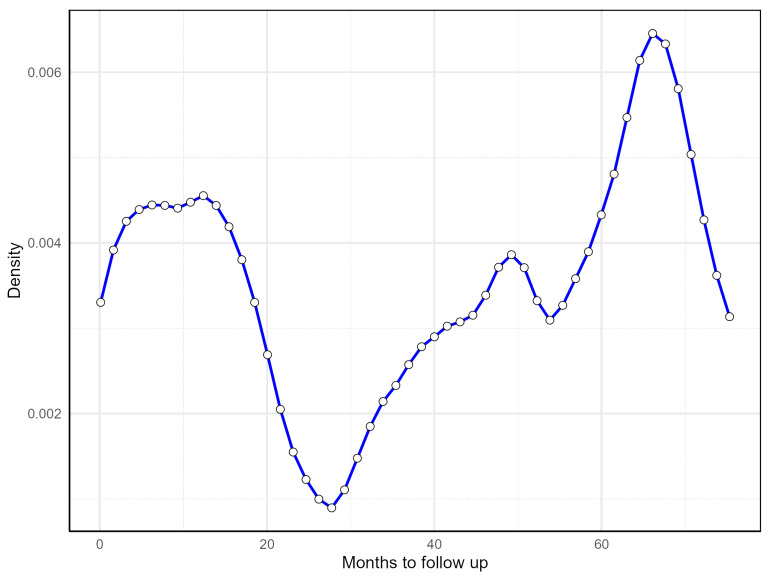
Fitted density of overall survival time in CRC patients (empty circles are observed survival times of CRC patients).

Recent studies emphasize the prognostic value of various DNA methylation biomarkers in CRC, including *LINE-1* [22], *CDKN2A* [23], and *IGFBP3* [24], as well as their combinations [25,26], though none have been used yet in routine clinical practice. Thus, there remains a critical need to discover and validate additional methylation prognostic biomarkers [27]. To find the most relevant methylated genes for CRC patient survival, two key factors must be addressed. First, hidden subpopulations of patients may introduce heterogeneity in the relationship between methylation and prognosis [28,29], as supported by our preliminary analysis (Figure 1). Second, considering the combined effects of multiple biomarkers is more beneficial than analyzing single markers [27]. However, not all methylation biomarkers may be significantly related to survival time in each subpopulation. To address these issues, a sparse estimation method in the finite mixture of accelerated failure time (FM-AFT) regression models [28] will be utilized.

The objective of this study is to develop an integrative framework for DMCs in cancer and assessing their prognostic significance. Using a penalized trans-dimensional hidden Markov model (HMM), we aim to enhance DMC detection while accounting for local autocorrelation and data sparsity. Additionally, a finite mixture of AFT regression model will be used to identify hidden subpopulations with distinct survival outcomes. Finally, functional enrichment analysis will evaluate the biological relevance of the identified DMCs, contributing to early detection and potential therapeutic target discovery.

The rest of the paper is organized as follows: Section 2 provides the materials, including the real datasets and the methods used for analyzing the data, such as the DMCTHM and fmrs methods for detecting important genes and their relationship with survival time. Section 3 presents a detailed analysis of the data and our interpretations. Section 4 offers a discussion of the results, followed by concluding remarks in Section 5.

## 2. Materials and Methods

This section describes the materials and proposed methods for data analysis. In Section 2.1, we present the datasets and preprocessing steps. Section 2.2 outlines the statistical methods and algorithms used for differential methylation detection. In Section 2.3, we describe the annotation of differentially methylated sites, focusing on genes with methylated CpGs in promoter regions.

Section 2.4 validates overlapping DMGs between CRC and ACF using six additional GEO datasets. To assess the functional relevance of identified methylated genes in early CRC and prognosis, we employ Gene Set Enrichment Analysis (GSEA) and Gene Ontology (GO) enrichment analysis (Section 2.5), identifying key biological processes, molecular functions, and cellular components associated with CRC progression [30]. These analyses aid in discovering potential therapeutic targets.

**Figure 2:**
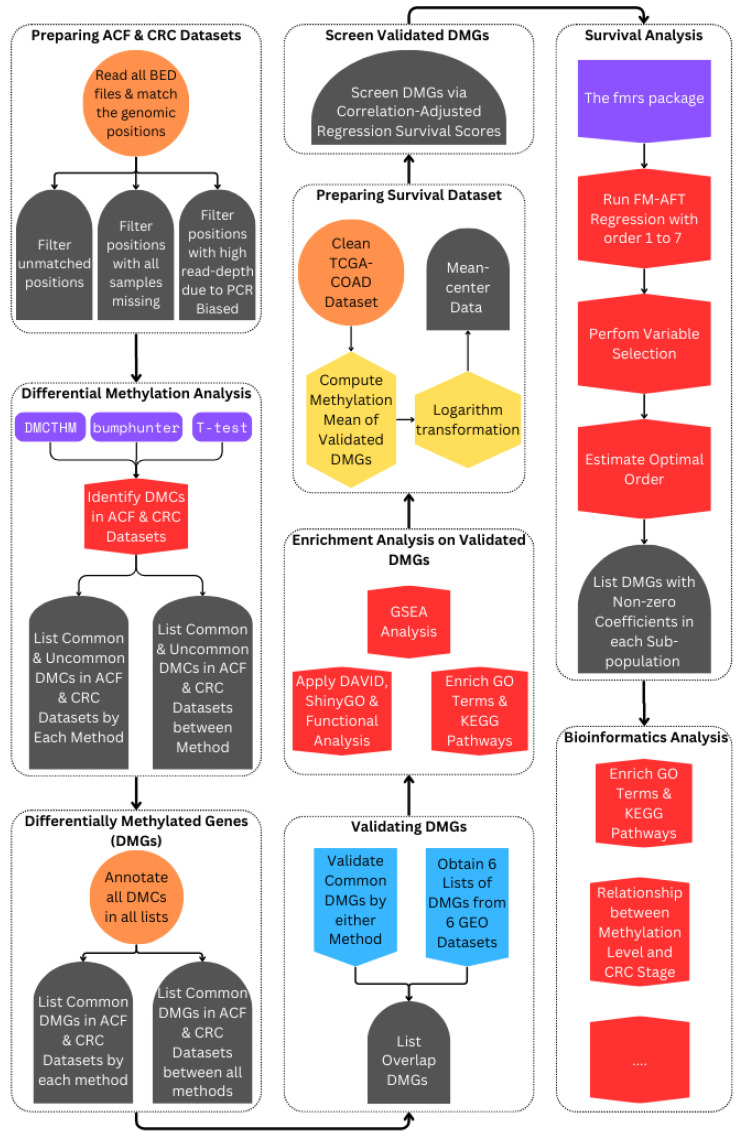
A flowchart of the study.

Finally, Section 2.6 introduces the FM-AFT regression model to assess the heterogeneity of DMGs’ effects on CRC patient survival. Figure 2 provides an overview of the study framework.

### 2.1. Colorectal Cancer Data

We acquired reduced-representation bisulfite sequencing (RRBS) data from the NCBI Gene Expression Omnibus (GEO, https://www.ncbi.nlm.nih.gov/geo/, accessed on 31 May 2024) accession number GSE95654 [16]. The GEO database, maintained by the National Center for Biotechnology Information (NCBI), is a publicly available repository that stores high-throughput gene expression, epigenomic, and other functional genomics datasets. It provides access to a wide range of studies, facilitating the exploration of molecular alterations across various biological conditions [31]. The acquired dataset consists of 10 samples of KRAS-mutated Stage III–IV CRC, along with their corresponding adjacent normal samples. Additionally, it includes 10 samples of KRAS-mutated ACF and their normal-appearing mucosa from the distal colon of individuals without familial adenomatous polyposis or hereditary non-polyposis CRC. To minimize the confounding effects of age and smoking on the association between DNA methylation and CRC, only non-smokers aged between 50 and 65 were included in the study. Following data processing of the 10 CRC and 10 ACF samples, along with their normal counterparts, and filtering out CpGs with extremely high read-depths (greater than 500) likely due to polymerase chain reaction bias, we retained data for 22,049,987 CpG dinucleotides.

**Figure 3:**
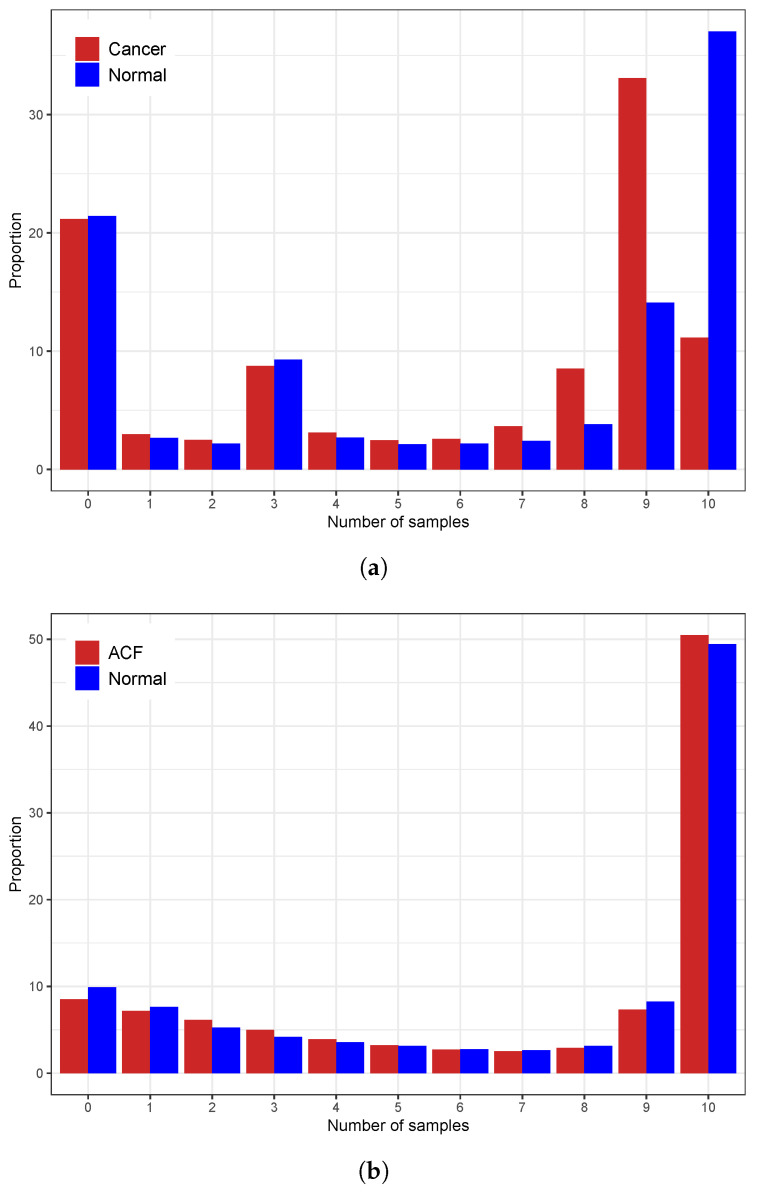
Proportion of missing values in (**a**) CRC and (**b**) ACF datasets.

The RRBS datasets exhibited a high rate of partial missing values, where some positions were sequenced in only a subset of samples. Figure 3 illustrates the distribution of missing values. In the ACF dataset, fewer than 10% of positions had complete data across all 20 samples, while in the CRC dataset, nearly 20% of CpGs had no missing information across all samples.

### 2.2. Differential Methylation Detection

The datasets were previously analyzed using *t*-tests to identify differential methylation [16]. However, the approach of the aforementioned study had several limitations that could affect the detection of DMCs. Firstly, they excluded all positions with partial missing values, resulting in significant data loss and introducing both statistical and biological biases, which adversely impact analyses such as gene selection and gene regulatory network studies [32,33,34]. Secondly, they set an arbitrary cutoff for read-depth at 5, a practice lacking consensus on the optimal minimum read-depth and primarily based on simulation studies without a clear theoretical rationale [35]. Moreover, the use of *t*-tests ignored key features of sequencing data, such as correlations among adjacent CpGs, which could lead to higher false positive rates [36]. The *t*-tests also focus solely on mean methylation ratios, disregarding read-depth, which affects the precision of the estimates [18]. Additionally, methylation data can contain outliers due to biological or technical variations, significantly affecting *t*-test results by skewing distributions and inflating variances, potentially leading to false positives or negatives in DMC detection [37].

To address these limitations, we analyzed the RRBS datasets using the DMCTHM [21] method and conducted a comparative analysis of our findings with those reported before in [16] and the R-package bumphunter [38].

DMCTHM assumes true methylated counts follow a beta-binomial distribution. It leverages the flexibility and interpretability offered by HMMs, using the correlation in methylation patterns across adjacent CpGs to smooth methylation levels. This approach allows for simultaneous estimation of HMM order and parameters using transdimensional Markov chain Monte Carlo, smoothing of methylation levels through penalization, and estimation of statistical uncertainty without imposing thresholds on read-depth or removing positions with partial missing values. We have previously demonstrated the superior performance of DMCTHM across various simulation scenarios, in which it outperformed more than 20 existing methods [21]. A brief description of DMCTHM follows.

Let $\left\{\left(y_{l},m_{l}\right),l=1,\ldots ,L\right\}$ represent the methylation read counts and read-depth at the $l^{\mathrm{th}}$ CpG site of a given sample. Read-depth refers to the total number of sequencing reads at a CpG site, while the methylation read count represents the number of methylated reads out of the total reads. DMCTHM assumes HMM$\left(S_{l},Y_{l}\right)$, where $S_{l}$ denotes the hidden state corresponding to methylation levels, and $Y_{l}$ is the read count conditional on $S_{l}$. The hidden states, {1,…,K}, represent distinct methylation levels, where each state *k* is associated with a methylation propensity $\theta_{k}$. Let ψ be the vector of all parameters. Given V=(y,s,m), the likelihood function is as follows:

L(ψ|V)=∏l=1Lmlyl∏k=1Kθk∑l:sl=kyl(1−θk)∑l:sl=k(ml−yl)∏k′=0Kpk′knk′k,

where $p_{k^{\prime }k}$ and $n_{k^{\prime }k}$ represent the transition probabilities and numbers between states, respectively. We developed a data-driven Bayesian approach to simultaneously estimate the HMM order and parameters. The joint *a priori* distribution is as follows:

$$\begin{array}{c} \pi \left(\psi \right)=\pi \left(K\right)\left\{\prod_{k=0}^{K}\pi \left(p_{k}|K\right)\right\}\left\{\prod_{k=1}^{K}\pi \left(\theta_{k}|K\right)\right\}, \end{array}$$

where $K∼\mathrm{Uniform}(1,\ldots ,K_{\max})$, for a pre-specified $K_{\max}$, $p_{k}|K∼\mathrm{Dirichlet}\left(\gamma_{k1},\ldots ,\gamma_{kK}\right)$, ∀k=0,1,…,K, $\theta_{k}|K∼\mathrm{Beta}\left(\alpha_{k},\beta_{k}\right)$, ∀k=1,…,K, and the hyper-parameters $\left(\gamma_{kK},\alpha_{k},\beta_{k}\right)$ are either fixed or sampled from a uniform distribution.

DMCTHM employs a reversible jump (RJ) algorithm. Initially, parameter values are updated using Gibbs sampling, followed by updating the HMM order through a split–merge move. The move is accepted based on the Metropolis–Hastings probability as follows:

$$\rho \left(\psi^{*}|\psi \right)=\min\left\{1,\frac{L(\psi^{*}|y,s^{*},m)}{L(\psi |y,s,m)}\frac{\pi (\psi^{*})}{\pi (\psi )}\frac{q(\psi |\psi^{*})}{q(\psi^{*}|\psi )}\right\},$$

where q(.|.) is the proposal distribution for drawing the movement.

To address under- and over-estimation in the HMM, two penalty functions are introduced: the first penalizes (increases) the HMM order in cases of under-estimation, while the second ensures that neighboring states collapse when they get very close to each other, addressing over-estimation.

After smoothing the methylation profiles for all subjects using the above method, DMCTHM applies a logit transformation to the averages of MCMC samples for each position and individual. A Bayesian linear model is then fitted to each CpG site, and *p*-values are acquired. Finally, DMCs are identified based on a false discovery rate (FDR) algorithm.

The data were analyzed using DMCTHM and bumphunter [38]. The lists of DMCs identified in CRC and ACF datasets were aligned to the human reference genome (GRCh37/19) using the UCSC Genome Browser (https://genome.ucsc.edu, accessed on 2 October 2024).

After identifying the DMCs, we used volcano plots to visualize the direction, magnitude, and statistical significance of methylation differences in CRC and ACF datasets. Each dot represents a CpG site, with those showing decreased fitted methylation levels (estimated via DMCTHM) appearing on the left side of the X-axis and those with increased levels on the right. CpGs with statistically significant methylation changes are located above the horizontal line at an FDR threshold of 0.05. Points near the center indicate minor changes, while those farther from the center reflect more substantial alterations.

### 2.3. Identifying Differentially Methylated Genes

Since tissue-specific promoter methylation impacts gene expression and, hence, the progression of the disease, we focused our analyses on these regions. The promoter regions were extracted using the R-packages TxDb.Hsapiens.UCSC.hg19.knownGene [39], org.Hs.eg.db [40], and annotate [41]. A total of 403 genes were dropped because they had exons located on both strands of the same reference sequence or on more than one reference sequence. Then, the genes with differentially methylated promoters (DMGs) were selected.

### 2.4. Validating the Detected Overlapping DMGs Between CRC and ACF

To gain deeper insights into genome-wide DNA methylation alterations in human ACF, we compared the identified DMGs in CRC and ACF using the findings of [16], the DMCTHM method, and the bumphunter R-package. Genes that overlapped in CRC and ACF datasets and were identified by either DMCTHM or the *t*-test formed our candidate gene list. This candidate list was then validated in other populations using the results of other studies.

For validation, we acquired six methylation datasets with accession numbers GSE42752 [42], GSE48684 [43], GSE53051 [44], GSE75546 [45], GSE77718 [46], and GSE101764 [47] from the GEO. The methyl array profiles of the validation sets were analyzed using the web tool GEO2R (http://www.ncbi.nlm.nih.gov/geo/geo2r/, accessed on 2 October 2024) and the R-package limma [48]. Probes were deemed differentially methylated if their adjusted *p*-values were below 0.05 and the absolute log_2_ (fold_change) in methylation was at least 0.1. These differentially methylated probes were subsequently aligned to the human reference genome (GRCh37/19) via the R-package FDb.InfiniumMethylation.hg19 [49]. A Venn diagram was constructed to compare the seven lists of identified DMGs and select the overlapping genes. The common genes, represented in the intersection, are considered validated DMGs.

### 2.5. Bioinformatics Analysis

We performed functional and pathway enrichment analysis using the database for annotation, visualization and integrated discovery (DAVID) [50] (https://david.ncifcrf.gov/, accessed on 2 October 2024) on the list of validated DMGs. GO terms and Kyoto Encyclopedia of Genes and Genomes (KEGG) [51] pathways were considered significantly enriched if FDR was less than 0.05. This analysis was done via the R-package clusterProfiler [52] and ShinyGO (Version 0.80) (http://bioinformatics.sdstate.edu/go/, accessed on 2 October 2024). To identify classes of genes that are over-represented in the DMGs list and may have an association with CRC, we utilized the R-package enrichplot [53] and the WEB-based Gene Set Analysis Toolkit (https://www.webgestalt.org, accessed on 2 October 2024) to implement GSEA.

### 2.6. Survival Analysis

To investigate the relationship between the methylation levels of validated DMGs and the survival time of CRC patients, prognostic data were selected from The Cancer Genome Atlas (TCGA). The data of 352 CRC patients were downloaded from the TCGA-COAD project [54]. Samples with missing follow-up times or patient status information were excluded, resulting in the removal of 100 samples. Preliminary analyses were conducted to select the appropriate models for handling right-censored data. The estimated density of survival times via the R-package survPresmooth [55] is depicted in Figure 1. In this plot, each dot represents the survival time of a CRC patient. The presence of multiple peaks suggests an underlying mixture distribution. This led to the hypothesis that the effect of identified DMGs on overall survival time varies across subpopulations. Additionally, it is plausible to assume a sparse regression model since only a handful of DMGs are assumed to have effects on survival time.

To capture this heterogeneity and sparsity, we applied the sparse estimation method in the finite mixture of AFT regression models with log-normal distributions [28]. The regression relationship in each mixture component is assumed to be

$$y^{*}=\log y=x\beta_{k}+\sigma_{k}\epsilon ,$$

where *y* is time-to-event data, x is the vector of DMGs, $\beta_{k}$ is the DMGs’ effects (some of which are zero), $\sigma_{k}^{2}$ is the variance of the *k*th component, and ϵ is the normal random error. The conditional likelihood of data is as follows:

$$\ell \left(\Psi \right)\propto \prod_{i=1}^{n}\sum_{k=1}^{K}\pi_{k}{\left[f(t_{i};x_{i}\beta_{k},\sigma_{k}^{2})\right]}^{\delta_{i}}{\left[S(t_{i};x_{i}\beta_{k},\sigma_{k}^{2})\right]}^{1-\delta_{i}},$$

where $t_{i}$ is the minimum of $y_{i}^{*}$ and censoring time $c_{i}$, $\delta_{i}$ is an indicator function for right-censoring ($\delta_{i}=0$ for censored data), $\pi_{k}$ ($\sum_{k=1}^{K}\pi_{k}=1$) gives the mixing probabilities, *f* and *S* denote the normal probability density and survival functions, respectively, and Ψ=(β,σ,π) is the vector of parameters.

To achieve sparsity, we penalized the regression model and obtained the penalized maximum likelihood estimator as follows:

$$\tilde{\Psi }=\arg\max\left\{\ell \left(\Psi \right)-n\sum_{k=1}^{K}\pi_{k}\sum_{j=1}^{d}p_{\lambda_{nk}}\left(\right|\beta_{kj}\left|\right)\right\}$$

by choosing the optimal tuning parameters $\lambda_{nk}$.

Note that we performed screening to reduce the number of DMGs and identified the most influential DMGs on the survival time of patients utilizing the correlation-adjusted scoring method via the carSurv R-package [56] prior to fitting the penalized regression model. This method is superior to univariate screening methods like univariate Cox regression, as it addresses the issue of correlation among DMGs. It applies a Mahalanobis-type transformation to de-correlate the DMGs, and then the correlation-adjusted regression survival (CARS) scores are computed. The most influential DMGs are selected based on the threshold of the 95th percentile of calculated CARS scores. The R-package fmrs [57,58] was then used to fit both penalized finite mixture and non-mixture AFT regression models to the data using the selected DMGs.

## 3. Results

In this Results section, the first phase of the study involved analyzing the RRBS data of CRC and ACF samples to identify DMCs, comparing them to matched normal mucosa samples using DMCTHM (Section 3.1). In Section 3.1.1, we compared our results with those from [16], obtained via *t*-test, and also reanalyzed the data using the R-package bumphunter [38]. After identifying the DMGs in each dataset (Section 3.2.1), we focused on DMGs related to the early stages of CRC by examining the overlap between the two datasets (Section 3.2.2). The validity of our findings was evaluated using several GEO datasets (Section 3.3). In Section 3.4, we conducted functional enrichment analysis of the validated DMGs. Finally, in Section 3.5, we identified validated DMGs whose methylation levels were associated with the survival of CRC patients.

### 3.1. Aberrantly Methylated CpG Sites

The DMCTHM method identified a total of 1,877,297 DMCs in the analysis of the CRC dataset, representing 8.5% of all CpGs. In contrast, the *t*-test method identified only 1.07% of CpGs as DMCs, and bumphunter detected a mere 0.02% of CpGs as DMCs in the CRC samples compared to adjacent normal samples. This result could imply that while [16] overlooked CpGs with low read-depth, the *t*-test was also unable to detect many DMCs with high read-depth. In the analysis of ACF vs. normal crypt samples, DMCTHM identified 0.5% of CpGs as DMCs, equating to 108,568 positions. The *t*-test and bumphunter identified 0.06% and 1% of CpGs as DMCs, respectively, by comparing ACF samples with their normal counterparts (Table 1).

**Figure 4:**
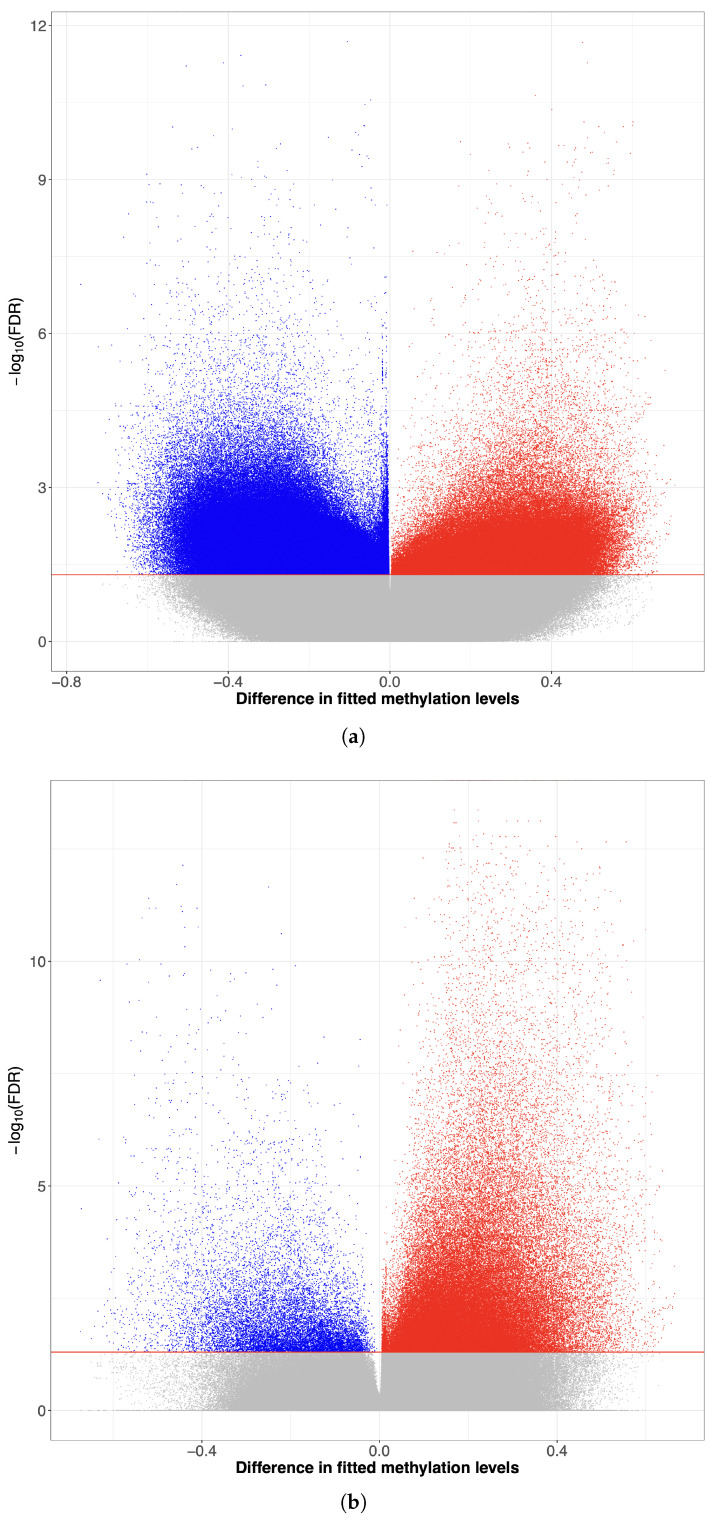
Volcano plot of predicted methylation of hypo-methylated DMCs (blue) and hyper-methylated DMCs (red) using DMCTHM. (**a**) CRC vs. adjacent normal colon samples. (**b**) ACF vs. normal crypt samples.

Based on Figure 4 and Supplementary Figure S1, it is evident that more of the DMCs identified by DMCTHM were hypo-methylated (88.9%) in CRC samples compared to their adjacent normal counterparts. On the other hand, Supplementary Figure S2 shows that the majority of DMCs identified by DMCTHM were hyper-methylated in ACF samples (87.9%) compared to normal crypt samples.

**Figure 5:**
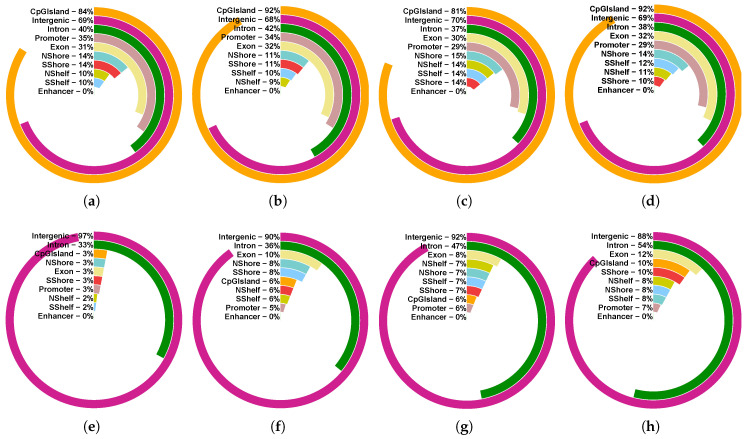
Genomic locations of identified hyper- (**a**–**d**) and hypo-methylated (**e**–**h**) DMCs in CRC (**a**,**b**,**e**,**f**) and ACF (**c**,**d**,**g**,**h**) datasets using DMCTHM (**a**,**c**,**e**,**g**) and *t*-test (**b**,**d**,**f**,**h**).

The distribution of the percentage of identified DMCs across various genomic contexts is depicted in Figure 5. The majority of hyper-methylated DMCs were located on CpG islands, while hypo-methylated DMCs were primarily found in intergenic regions, introns, and exons. For the *t*-test, the proportion of identified hypo-methylated DMCs in intergenic regions was lower compared to DMCTHM, possibly due to low read-depth within these regions.

#### 3.1.1. Agreement Between DMCTHM, *t*-Test, and bumphunter in Detecting DMCs

Notably, DMCTHM captured 27% and 60% of the DMCs identified by the *t*-test, and 9% and 0.26% of the DMCs identified by bumphunter in the CRC and ACF datasets, respectively (Table 2 and Table 3). Promoters were the genomic locations showing the highest agreement between the *t*-test and DMCTHM in detecting DMCs, whereas shelves showed the lowest agreement. Meanwhile, bumphunter detected no DMCs in promoters and CpG Islands.

Supplementary Tables S1 and S2 and Figures S1 and S2 show the proportion of identified DMCs in each chromosome. Notably, both DMCTHM and the *t*-test identified Chromosomes 20, 18, 7, 4, and 8 as the most methylated during the progression of CRC; however, the *t*-test identified a relatively small number of DMCs in Chromosomes 13 and X compared to DMCTHM. Furthermore, the identified DMCs in Chromosomes 1, 2, and 6 via DMCTHM provide stronger evidence of differential methylation patterns in ACF samples compared to normal crypt samples.

Supplementary Table S3 presents the direction of identified DMCs in all CpGs, stratified by genomic locations in the CRC dataset. When both methods identify a CpG as a DMC, they mostly (99.7%) agree on the direction of methylation. Importantly, the majority of DMCs solely detected by DMCTHM exhibited hyper-methylation in CpG islands, promoters, and exons, while showing hypo-methylation in intergenic regions, introns, shores, and shelves.

Both DMCTHM and the *t*-test identified CpG islands and intergenic regions as the most prevalent genomic locations of common hyper-methylated DMCs in the CRC and ACF datasets. However, DMCTHM found more common hyper-methylated DMCs in promoters compared to the *t*-test.

### 3.2. Aberrantly Methylated Genes

Recognizing the crucial role of promoter methylation in the development and progression of CRC [59], our subsequent analysis concentrates on DMCs within gene promoters.

#### 3.2.1. Identifying Hypo/Hyper DMGs in CRC and ACF

In the CRC dataset, we identified a total of 6410 DMGs using DMCTHM (1651 hyper-methylated and 4759 hypo-methylated DMGs) and a total of 1886 DMGs (1104 hyper-methylated and 782 hypo-methylated DMGs) using the *t*-test. Out of the 1886 DMGs identified by the *t*-test, 1388 (73%) were also detected by DMCTHM.

**Figure 6:**
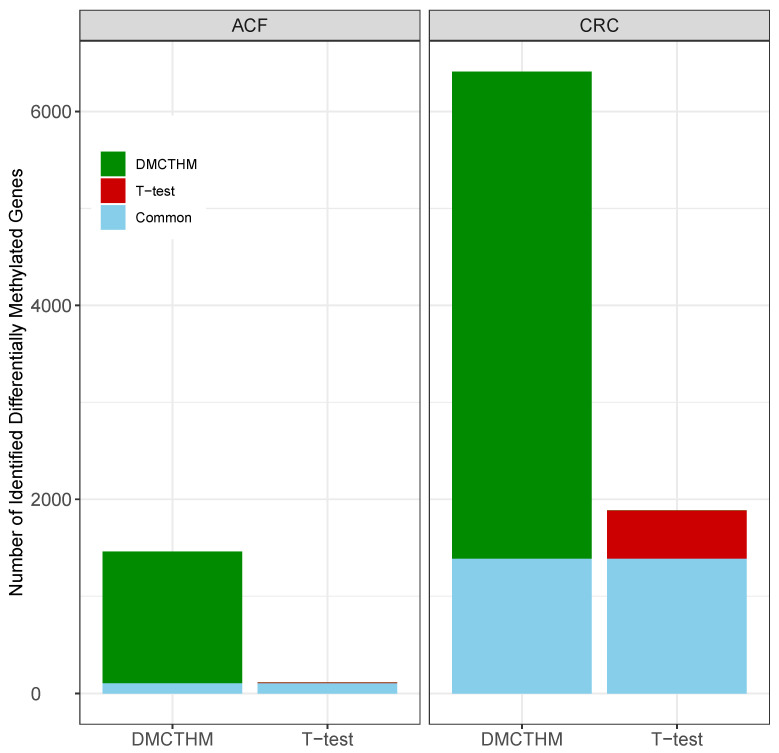
Differentially methylated gene distribution via DMCTHM and *t*-test.

In the ACF dataset, we identified 1462 DMGs (1222 hyper-methylated and 240 hypo-methylated DMGs), while 105 hyper-methylated DMGs and 11 hypo-methylated DMGs were identified by the *t*-test. Notably, DMCTHM identified 92% of the DMGs detected by the *t*-test, including 106 hyper-methylated and 6 hypo-methylated DMGs (Figure 6). Since bumphunter did not identify any DMCs within gene promoters, no further analysis was performed.

#### 3.2.2. Overlapping DMGs Between CRC and ACF

Using DMCTHM, we identified a total of 37,022 overlapping DMCs between the CRC and ACF datasets. Among these DMCs, 35,568 exhibited consistent methylation patterns, with 32,960 (92.7%) being hyper-methylated and 2608 (7.3%) hypo-methylated. Notably, these co-occurring hyper- and hypo-methylated DMCs were situated within the promoter regions of 660 and 86 genes, respectively. Furthermore, 173 DMGs were hypo-methylated in CRC while hyper-methylated in ACF and 15 DMGs were hypo-methylated in ACF but hyper-methylated in the later stages of CRC.

Conversely, the *t*-test analysis revealed 10,724 overlapping DMCs between the CRC and ACF datasets. Among these, 10,143 DMCs displayed consistent methylation patterns, with 9014 (88.9%) being hyper-methylated and 1129 (10.1%) hypo-methylated. Specifically, the hyper-methylated DMCs were located within the promoters of 94 genes, while hypo-methylated DMCs were identified within the promoters of 6 genes. Notably, DMCTHM successfully identified 84% of the overlapping DMGs between CRC and ACF detected by the *t*-test. Additionally, DMCTHM identified 850 genes with differentially methylated promoters in both the CRC and ACF datasets compared to their normal counterparts.

### 3.3. External Validity Using GEO Datasets

**Figure 7:**
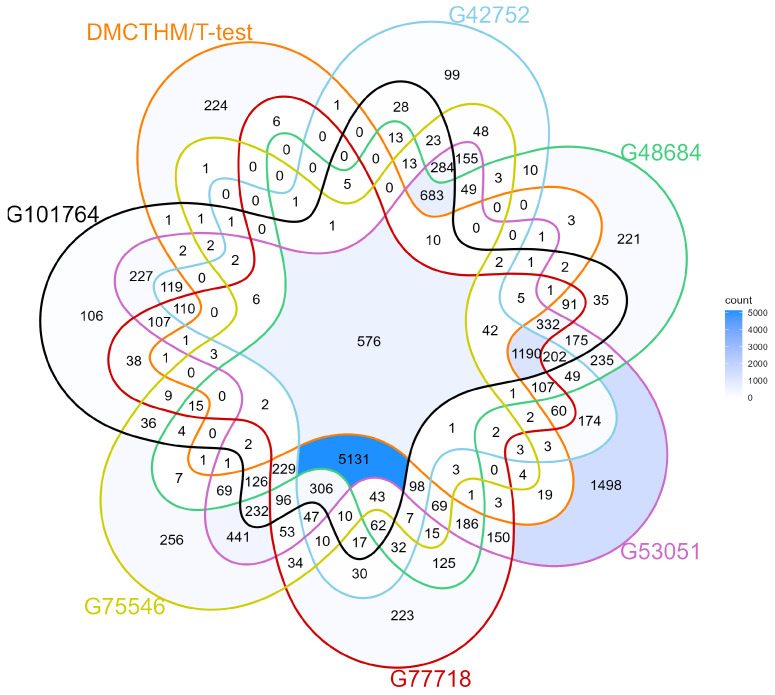
Venn diagram of commonly identified DMGs in CRC and ACF datasets using DMCTHM, *t*-test, and GEO datasets.

Our final list of DMGs comprises 950 genes, with 850 uniquely identified by DMCTHM, 16 identified exclusively by the *t*-test, and the remainder identified by both DMCTHM and the *t*-test. To validate these findings, we conducted an intersection with DMGs identified from selected GEO datasets comparing CRC and normal samples (Figure 7). This analysis revealed that 576 genes (of 950 genes) were consistently methylated across the various GEO datasets, of which 502 (of 850 genes) were identified only by DMCTHM. Consequently, we focused our subsequent analysis on this validated subset of DMGs to gain a more comprehensive understanding of their role in early CRC pathogenesis.

### 3.4. Functional Enrichment Analysis of Validated DMGs

Promoter methylation is involved in the regulation of various signaling pathways implicated in CRC development. The KEGG pathway analysis (Table 4) revealed that DMGs were significantly enriched in the calcium signaling pathway, synaptic vesicle cycle, neuroactive ligand–receptor interaction pathway, ECM–receptor interaction, and protein digestion and absorption.

**Figure 8:**
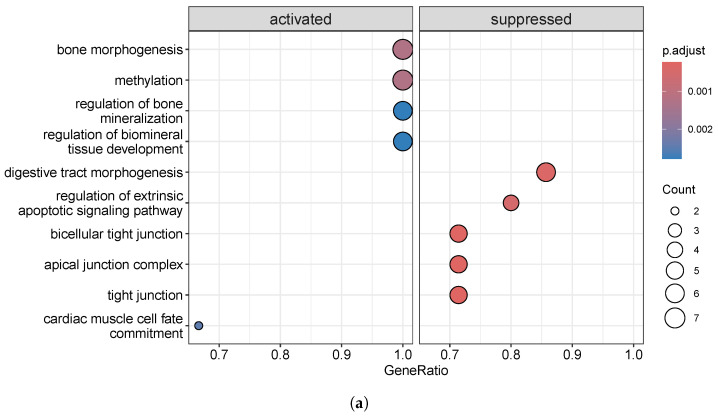

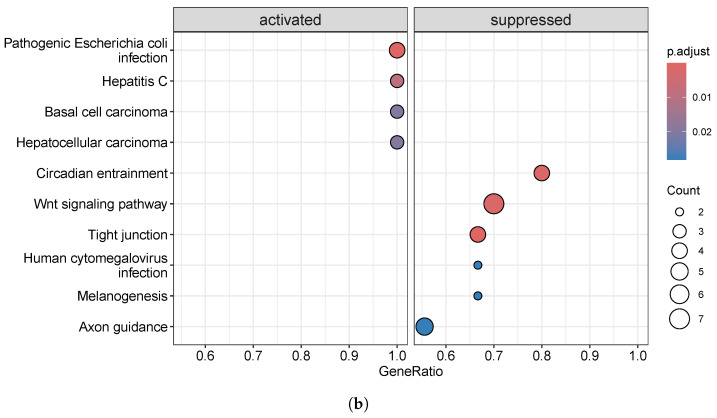
Gene set enrichment analysis of overlapped DMGs in CRC/ACF datasets identified by DMCTHM and *t*-test: (**a**) Gene Ontology; (**b**) KEGG Pathway.

The GO analysis (Figure 8) showed that changes in biological processes were significantly enriched in spinal cord development, cell fate commitment, and sensory organ morphogenesis, among others. Among the enriched cellular components based on the list of identified DMGs, the potassium channel complex, presynaptic membrane, intrinsic component of the postsynaptic membrane, and integral component of the postsynaptic membrane are the most important ones. Moreover, molecular functions were significantly enriched in voltage-gated cation channel activity, potassium ion transmembrane transporter activity, cation channel activity, and gated channel activity.

### 3.5. Role of Validated DMGs in Survival Time of Patients

To evaluate the relationship between the methylation of genes in the early stages of CRC and the overall survival of patients, we conducted a survival analysis using 252 samples from the TCGA-COAD dataset.

In the initial step of screening for influential DMGs, the correlation-adjusted survival scores were computed. DMGs with strong correlations to overall survival time were selected by applying a threshold to the ranked CARS scores, with the cutoff set at the 95th percentile. This process yielded a list of 19 DMGs: *CDH11*, *FOXF1*, *TRIM29*, *DCHS2*, *TMEM215*, *GALNT13*, *MIR34B*, *CHST10*, *TFAP2B*, *EPB41L3*, *DOCK2*, *SLC4A11*, *PPP1R14A*, *GPR158*, *TFAP2C*, *STX18*, *RAMP3*, *MEF2D*, and *NAPSB*. Notably, 16 of these were solely identified by DMCTHM, while the *t*-test detected just one of them.

To account for the heterogeneity in the data, as evident from the estimated density of survival times (Figure 1), we analyzed the data using a sparse FM-AFT regression models via the R-package fmrs. In this model, the covariates included a matrix of mean-adjusted logarithms of promoter methylation for the list of 19 DMGs, with the overall survival times of CRC patients treated as the right-censored dependent variable. To determine the appropriate number of mixture components in the data, we fitted various FM-AFT regression models with K=1,…,7. The model with K=2 components had the lowest BIC and was selected as the final model.

**Figure 9:**
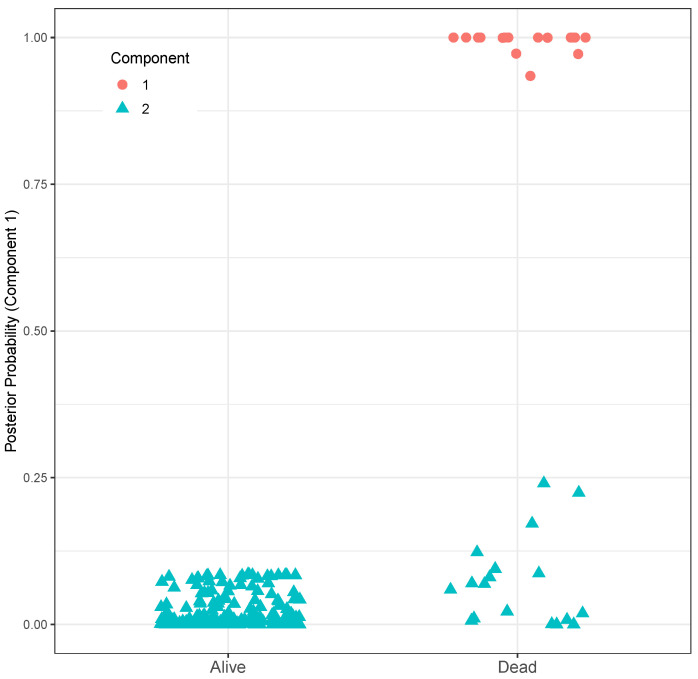
Posterior probabilities of patients belonging to Component 1, with *Alive* and *Dead* patients separated.

The estimated coefficients of methylation levels of the 19 genes for survival time in each subpopulation are presented in Table 5. Under this model, patients in Component 1 exhibited the shortest survival times, while 92% of patients in Component 2 had the longest survival times (Supplementary Figure S3). Based on the posterior probabilities of Component 1 (Figure 9), all surviving CRC patients were categorized into Component 2. This indicates that patients belonging to Component 1 experience a more aggressive form of the disease, while those in Component 2 exhibit a less aggressive form of CRC. Seven genes, including *CDH11*, *TMEM215*, *EPB41L3*, *DOCK2*, *PPP1R14A*, *GPR158*, and *NAPSB*, were associated with survival time in one of the components. Four of them were active in Component 2, and a total of three DMGs were active in Component 1. Notably, none of the active genes in the two components overlapped. The underlying functional profiles of this set of genes are illustrated in Supplementary Figure S4. These findings demonstrate the heterogeneity of DMG effects in CRC data and justify the use of sparse mixture modeling rather than a univariate model. Furthermore, the DMGs with active promoters in Component 1 can be considered potential biomarkers for the early diagnosis of CRC (Supplementary Figure S5).

## 4. Discussion

In this study, we employed advanced statistical and machine learning techniques, notably DMCTHM for detecting DMCs and DMGs related to CRC and ACF, and fmrs for sparse estimation in FM-AFT regression models. These methodologies effectively address key challenges in DNA methylation analysis and complex regression modeling, offering valuable insights into the early detection and progression of CRC.

Using DMCTHM, we identified eight-fold more DMCs compared to the *t*-test [16] and bumphunter in both CRC and ACF datasets. Further analyses revealed that while [16] overlooked CpGs with low read-depth, many undetected DMCs by the *t*-test have reasonably large read-depth. This may imply the insufficient statistical power of such naive tests. These DMCs predominantly exhibited hyper-methylation in CpG islands, promoters, and exons, aligning with studies linking CpG island hyper-methylation to tumor suppressor gene silencing [60]. Hypo-methylation was also observed in intergenic regions, introns, shores, and shelves, with a significant subset of DMCs mapped to Chromosome 13, a locus recurrently altered in CRC [61].

Further investigation into the methylation patterns of late-stage CRC and ACF samples revealed a notable contrast. Late-stage CRC predominantly exhibited hypo-methylation, particularly in intergenic regions, consistent with previous research suggesting a global loss of DNA methylation during tumor progression [16,62]. In contrast, ACF samples showed hyper-methylation. This discrepancy between ACF and CRC highlights the role of overall methylation status shifts in the progression from early neoplasia to invasive CRC.

Although [16] reported a high prevalence of hyper-methylation in DMC identification, our findings suggest that DMCTHM detected a greater number of hyper-methylated DMCs within the promoter of 950 genes, further highlighting its effectiveness in pinpointing functionally significant methylation events, as supported by other studies [63].

Pathway enrichment analysis revealed significant associations between many identified DMGs and CRC-related pathways, including synaptic vesicle cycle, ECM–receptor interaction, calcium signaling, protein digestion and absorption, neuroactive ligand–receptor interaction, cAMP signaling, and PI3K-Akt signaling pathways. Notably, the neuroactive ligand–receptor interaction pathway, involved in crucial intracellular and extracellular signaling, has been implicated in CRC [64]. These findings align with previous studies [65,66], emphasizing the relevance of our identified DMGs in CRC pathogenesis.

Beyond pathway enrichment, we also identified genes associated with CRC patient survival. FM-AFT regression analysis indicated a two-component model, with genes such as *CDH11*, *EPB41L3*, and *DOCK2* associated with more aggressive forms of CRC, while *TMEM215*, *PPP1R14A*, *GPR158*, and *NAPSB* were linked to less aggressive forms. These genes were not detected by conventional methods, demonstrating the unique strengths of our approach in identifying clinically relevant markers. Among these, *CDH11*, a cadherin family member located on Chromosome 16q22.1, emerged as a potential tumor suppressor in CRC. Its down-regulation is linked to reduced cell cycle arrest, apoptosis, and increased tumor cell proliferation, migration, and invasion [67]. *CDH11* exerts tumor-suppressive effects through pathways such as Wnt/β-catenin, AKT/Rho A, and NF-*κ*B [68]. Similarly, *EPB41L3*, a membrane skeletal protein involved in cell adhesion, has demonstrated tumor-suppressive effects across various cancers, including CRC [69,70]. Additionally, *DOCK2*, primarily expressed in hematopoietic cells, plays a key role in immune cell function, with its overexpression linked to improved prognosis and greater CD8+ T cell infiltration in CRC [71]. Furthermore, *PPP1R14A*, known for its epigenetic regulation in CRC, was down-regulated in tissue samples but up-regulated in CRC cell lines after 5-aza treatment, highlighting its potential as an epigenetically regulated gene [72,73].

Collectively, these findings provide new insights into the molecular mechanisms driving CRC progression and highlight potential targets for methylation-based therapeutic strategies. DNA methylation and histone acetylation influence nucleosome positioning by modulating the electrostatic interactions between DNA and histones. While methylation does not directly alter DNA’s charge, it can affect the strength of histone–DNA interactions, impacting nucleosome stability and positioning [74]. Histone acetylation further reduces electrostatic attraction, modifying chromatin structure. Integrating these theoretical concepts with our data analysis enables a more comprehensive understanding of how DNA modifications affect nucleosome positioning.

From a translational perspective, our findings have significant public health and clinical implications. Governments can prioritize funding for large-scale validation of DNA methylation biomarkers (e.g., shared DMCs/DMGs between CRC and ACF) and incorporate non-invasive methylation-based tests into national screening programs. Healthcare policymakers can update clinical guidelines to adopt advanced methylation-based prognostic models, such as FM-AFT or DMCTHM, to facilitate precision medicine strategies. At the community level, increasing awareness of early CRC screening and non-invasive testing can help reduce CRC mortality and healthcare costs through early intervention.

Despite these promising results, our study has some limitations. The datasets we analyzed did not include information on potential confounding variables that could affect methylation patterns, although our method is capable of incorporating such variables into the model. However, validation of our identified DMGs using other datasets helps mitigate this limitation.

## 5. Conclusions

By utilizing our proposed DMCTHM and sparse FM-AFT regression methods, we introduced an efficient protocol for identifying key biomarkers related to early CRC detection and prognosis. Our approach uncovered many shared DMGs between CRC and ACF, with several uniquely detected by DMCTHM, emphasizing its ability to capture biologically significant new methylated promoters. The observed hyper-methylation in ACF, along with the predominant hypo-methylation in late-stage CRC, aligns with established tumorigenesis mechanisms. This shifting methylation status could be seen as an epigenetic mark for monitoring neoplastic transition. Additionally, pathway enrichment analysis linked key DMGs to CRC-related signaling pathways, while FM-AFT regression identified novel prognostic genes such as *CDH11*, *EPB41L3*, and *DOCK2*, highlighting the clinical relevance of our findings. Particularly, genes that are active in the aggressive CRC subtype provide actionable and therapeutic targets and can help with risk stratification.

Our results pave the way for new directions in epigenetic biomarker discovery and precision oncology. Furthermore, integrating methylation-based prognostic models into clinical decision-making could enhance personalized treatment strategies by identifying patients at higher risk of disease progression and enabling deep insights into CRC’s epigenetic evolution.

## Figures and Tables

**Table 1:** Comparison of percentage of identified DMCs using DMCTHM, *t*-test, and bumphunter.

	DMCTHM		*t*-test		bumphunter	
Dataset	#DMCs	%DMCs	#DMCs	%DMCs	#DMCs	%DMCs
CRC	1,877,297	8.51	235,692	1.07	4530	0.020
ACF	108,568	0.49	12,752	0.06	1557	0.007

**Table 2:** Comparison of identifying DMCs in colorectal data: DMCTHM vs. *t*-test.

Dataset		*t*-test					
		NDMC	% of 2 × 2 Table	% of *t*-Test NDMC	DMC	% of 2 × 2 Table	% of *t*-Test DMC
CRC							
All							
DMCTHM	NDMC	19,999,661	90.70		173,029	0.78	
	DMC	1,814,634	8.23	8.32	62,663	0.28	26.59
Island							
DMCTHM	NDMC	3,255,545	91.68		76,337	2.15	
	DMC	184,822	5.20	5.37	34,411	0.97	31.07
Promoter							
DMCTHM	NDMC	2,324,726	93.96		30,639	1.24	
	DMC	105,107	4.25	4.33	13,571	0.55	30.70
ACF							
All							
DMCTHM	NDMC	21,936,172	99.48		5241	0.02	
	DMC	101,063	0.46	0.46	7505	0.03	58.88
Island							
DMCTHM	NDMC	3,470,005	97.72		3173	0.09	
	DMC	71,461	2.01	2.02	6470	0.18	67.10
Promoter							
DMCTHM	NDMC	2,444,256	98.80		1040	0.04	
	DMC	26,690	1.08	1.08	2057	0.08	66.42

**Table 3:** Comparison of identifying DMCs in colorectal data: DMCTHM vs. bumphunter.

Dataset		bumphunter					
		NDMC	% of 2 × 2 Table	% of bumphunter NDMC	DMC	% of 2 × 2 Table	% of bumphunter DMC
CRC							
All							
DMCTHM	NDMC	20,168,568	91.47		4122	0.02	
	DMC	1,876,889	8.51	8.51	408	0.002	9
Island							
DMCTHM	NDMC	3,331,000	93.80		882	0.02	
	DMC	219,179	6.17	5.77	54	0.001	6.17
Promoter							
DMCTHM	NDMC	2,354,715	95.18		650	0.03	
	DMC	118,663	4.80	4.80	15	0.0006	2.25
ACF							
All							
DMCTHM	NDMC	21,939,860	99.50		1553	0.007	
	DMC	108,564	0.49	0.49	4	0.00002	0.26
Island							
DMCTHM	NDMC	3,472,930	97.79		248	0.007	
	DMC	77,931	2.19	2.19	0	0.0	0.0
Promoter							
DMCTHM	NDMC	2,445,152	98.83		144	0.006	
	DMC	28,747	1.16	1.16	0	0.0	0.0

**Table 4:** KEGG pathway over-representation analysis of overlapped DMGs in CRC and ACF datasets identified by DMCTHM and *t*-test.

Enrichment FDR	nGenes	Pathway Genes	Fold Enrichment	Pathway	Matching Proteins in Network (Labels)
0.01	8	78	4.96	Synaptic vesicle cycle	RIMS1, SLC6A11, SNAP25, CACNA1A, CACNA1B, SLC18A2, SLC18A3, DNM3
0.02	8	88	4.40	ECM–receptor interaction	ITGA8, COL9A3, LAMA1, COL4A2, COL6A2, AGRN, RELN, LAMA2
0.00002	20	240	4.03	Calcium signaling pathway	FLT4, FGF4, NTSR1, FGF6, HRH2, ADRA1A, LHCGR, FGF5, NTRK3, CACNA1A, CACNA1B, PDE1C, ATP2B2, PTGER1, GDNF, DRD5, ERBB4, FGF3, CACNA1H, RYR3
0.04	8	103	3.76	Protein digestion and absorption	ELN, COL9A3, COL5A1, COL4A2, COL6A2, COL24A1, COL18A1, COL25A1
0.00002	25	350	3.46	Neuroactive ligand–receptor interaction	ADCYAP1R1, SCTR, CHRNA3, OPRK1, NTSR1, OXT, VIPR2, GLP1R, HRH2, GRIN3B, CNR1, ADRA1A, SSTR4, LHCGR, NPFFR1, GRIA4, PTGER1, GRIK3, GRIK2, DRD5, SSTR2, GRID1, GRM7, S1PR3, NPBWR1
0.01	14	219	3.09	CAMP signaling pathway	ADCYAP1R1, OXT, PDE4C, VIPR2, GLP1R, PDE10A, GRIN3B, VAV3, LHCGR, HCN4, GRIA4, ATP2B2, DRD5, SSTR2
0.04	17	354	2.32	PI3K-Akt signaling	FLT4, FGF4, ITGA8, COL9A3, TCL1A, LAMA1, FGF6, COL4A2, FGF5, PIK3R5, COL6A2, EIF4E1B, ERBB4, FGF3, RELN, LAMA2, TCL1B

**Table 5:** Estimated DMG effects in two-component mixture of accelerated failure time regression model for TCGA-COAD data.

Coefficients	$\beta_{1j}$	$\beta_{2j}$
Intercept	1.83	5.48
*CDH11*	−3.60	0.00
*FOXF1*	0.00	0.00
*TRIM29*	0.00	0.00
*DCHS2*	0.00	0.00
*TMEM215*	0.00	−3.08
*GALNT13*	0.00	0.00
*MIR34B*	0.00	0.00
*CHST10*	0.00	0.00
*TFAP2B*	0.00	0.00
*EPB41L3*	2.11	0.00
*DOCK2*	−3.71	0.00
*SLC4A11*	0.00	0.00
*PPP1R14A*	0.00	−2.36
*GPR158*	0.00	1.86
*TFAP2C*	0.00	0.00
*STX18*	0.00	0.00
*RAMP3*	0.00	0.00
*MEF2D*	0.00	0.00
*NAPSB*	0.00	−2.93

## Data Availability

In this study, methylation profiling datasets with accession numbers GSE95656 [16], GSE42752 [42], GSE48684 [43], GSE53051 [44], GSE75546 [45], GSE77718 [46], and GSE101764 [47] were obtained from GEO at the NCBI. Moreover, the TCGA-COAD dataset was obtained from the Genomic Data Commons (GDC) Data Portal [75].

## Supplementary Materials

The following supporting information can be downloaded at: https://www.mdpi.com/article/10.3390/biom15020294/s1. The list of final validated DMGs (DMGlist.csv) and the Web-based Supplement (WebSup.pdf) are available on the *Biomolecules* website. Tables S1 and S2 present the percentage of CpGs identified as DMCs by DMCTHM in CRC and ACF across each chromosome. Table S3 provides a cross-tabulation of the direction of methylation (hypo-methylated or hyper-methylated) of cytosines identified by DMCTHM and *t*-test, categorized by genomic location. Figures S1 and S2 display Manhattan plots of DMCs identified by DMCTHM for the CRC and ACF datasets, respectively. Figure S3 shows the density of the logarithm of fitted values in the survival model. Figure S4 illustrates the enriched biological processes, molecular functions, and pathways associated with survival-related DMGs. Figure S5 shows the DNA methylation levels of genes related to survival time, categorized by CRC stages.

## Abbreviations

The following abbreviations are used in this manuscript:

ACF	Aberrant Crypt Foci
CARS	Correlation-Adjusted Regression Survival
CRC	Colorectal Cancer
DAVID	Database for Annotation, Visualization, and Integrated Discovery
DMC	Differentially Methylated Cytosine
DMCTHM	Differentially Methylated Cytosine Based on Trans-Dimensional Hidden Markov Model
DMG	Differentially Methylated Gene
FDR	False Discovery Rate
FM-AFT	Finite Mixture of Accelerated Failure Time
GDC	Genomic Data Commons
GEO	Gene Expression Omnibus
GO	Gene Ontology
GSEA	Gene Set Enrichment Analysis
HMM	Hidden Markov Model
KEGG	Kyoto Encyclopedia of Genes and Genomes
RJ	Reversible Jump
RRBS	Reduced-Representation Bisulfite Sequencing
TCGA	The Cancer Atlas Genome
