# Supplementary material for: Data-Driven Identification of Early Cancer-Associated Genes via Penalized Trans-Dimensional Hidden Markov Models
Source: Biomolecules. 2025 Feb 16;15(2):294. doi: 10.3390/biom15020294 (PMC11853217; doi:10.3390/biom15020294)
Supplement: Supplementary file 1 [file biomolecules-15-00294-s001.zip › biomolecules-3456209-supplementary.pdf]

Web-based Supplementary Materials for "Data-Driven Identification of Early Cancer-Associated Genes via Penalized Trans-dimensional Hidden Markov Models"

Saeedeh Hajebi Khaniki
Department of Biostatistics, Mashhad University of Medical Sciences, Mashhad, Iran
and
Farhad Shokoohi
Department of Mathematical Sciences, College of Sciences
University of Nevada, Las Vegas, Las Vegas, NV 89154, USA

February 13, 2025

Abstract

In this Web-Supplement, we present the materials referred to in the main paper. The contents are as follows:

Contents

S1 Figures **2**

S2 Tables **7**

S1 Figures

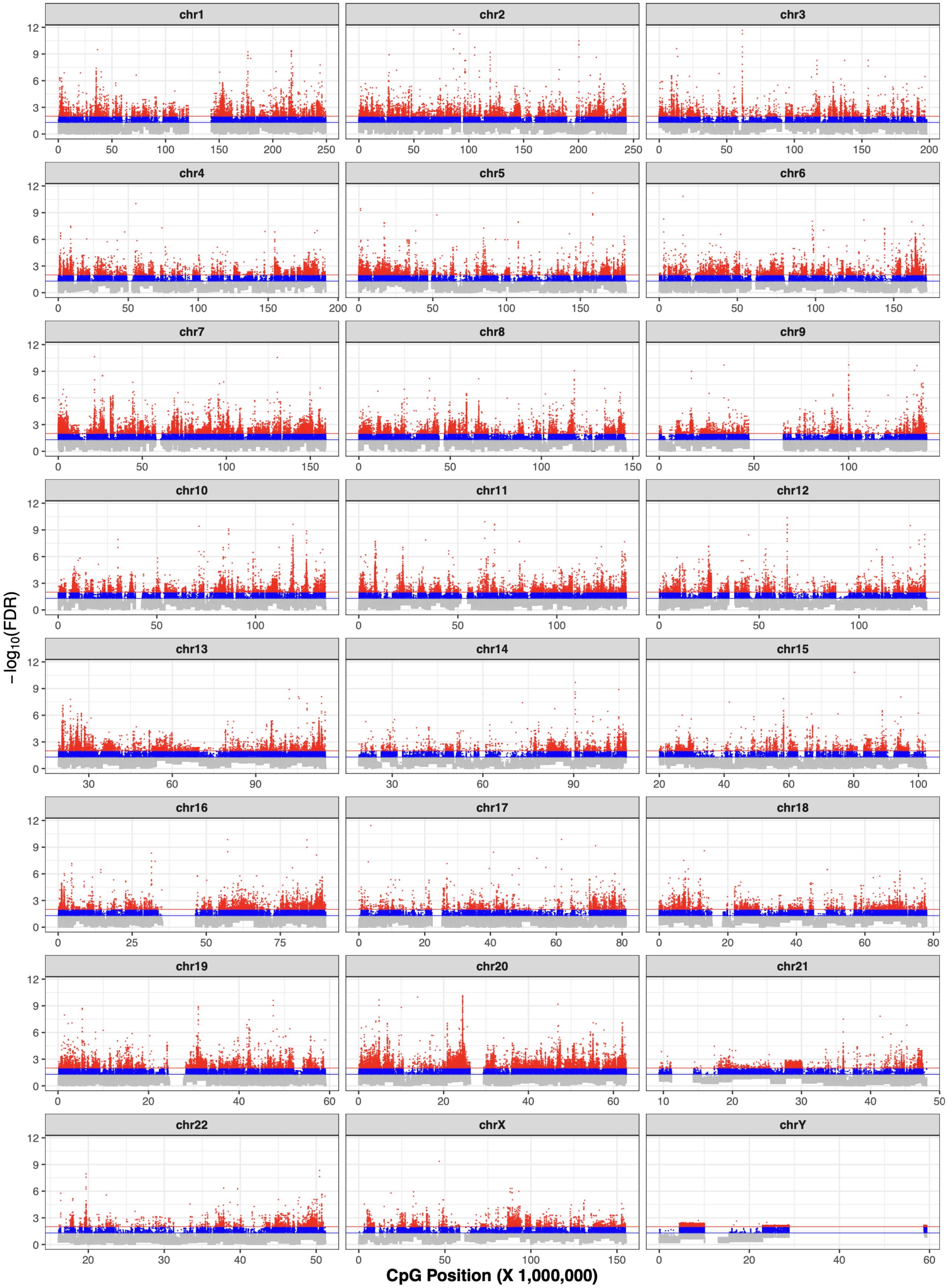

Figure S1: Manhattan plot of the CRC dataset. Strong significance is depicted in red.

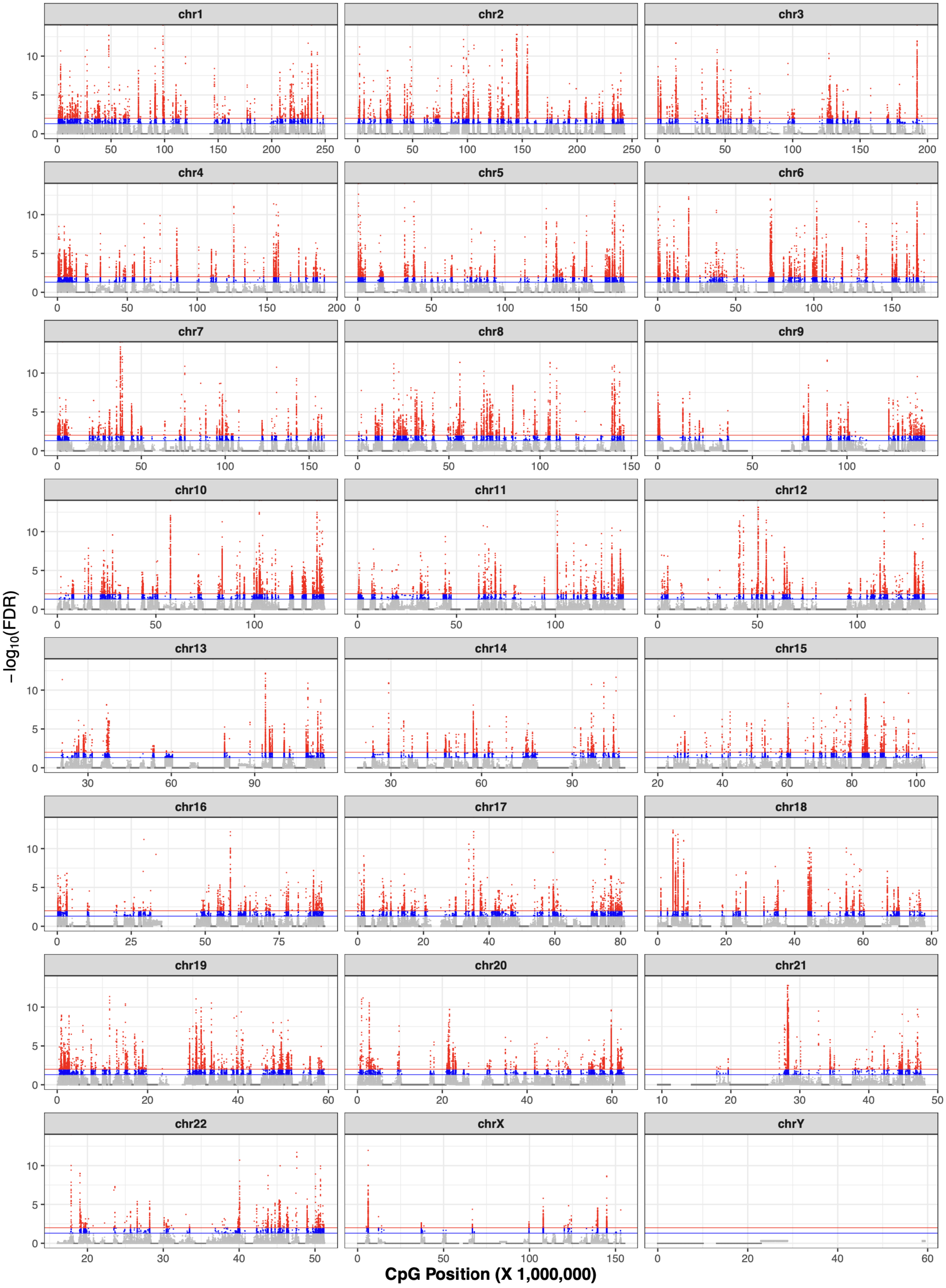

Figure S2: Manhattan plot of the ACF dataset. Strong significance is depicted in red.

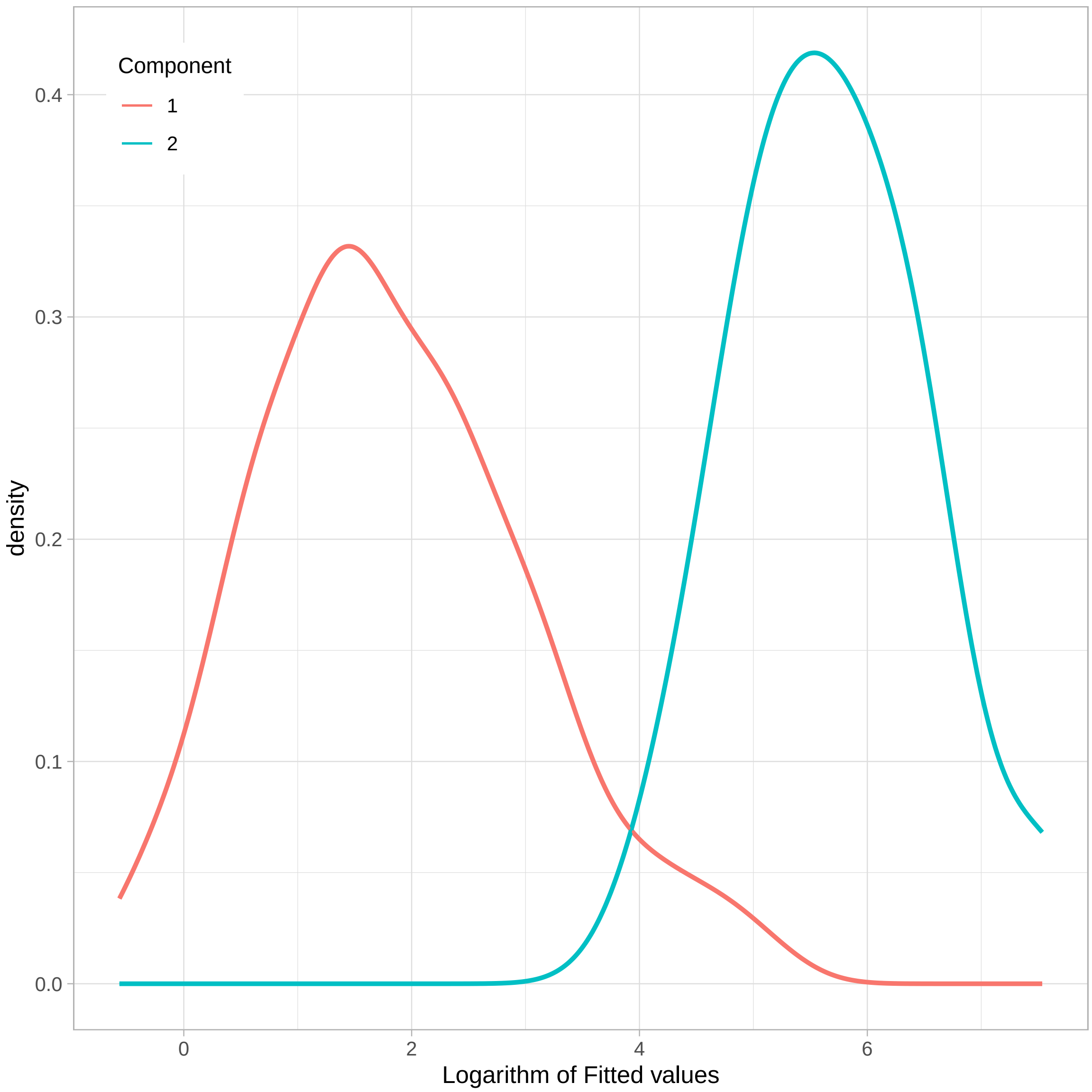

Figure S3: Density of logarithm of fitted values for the TCGA-COAD data.

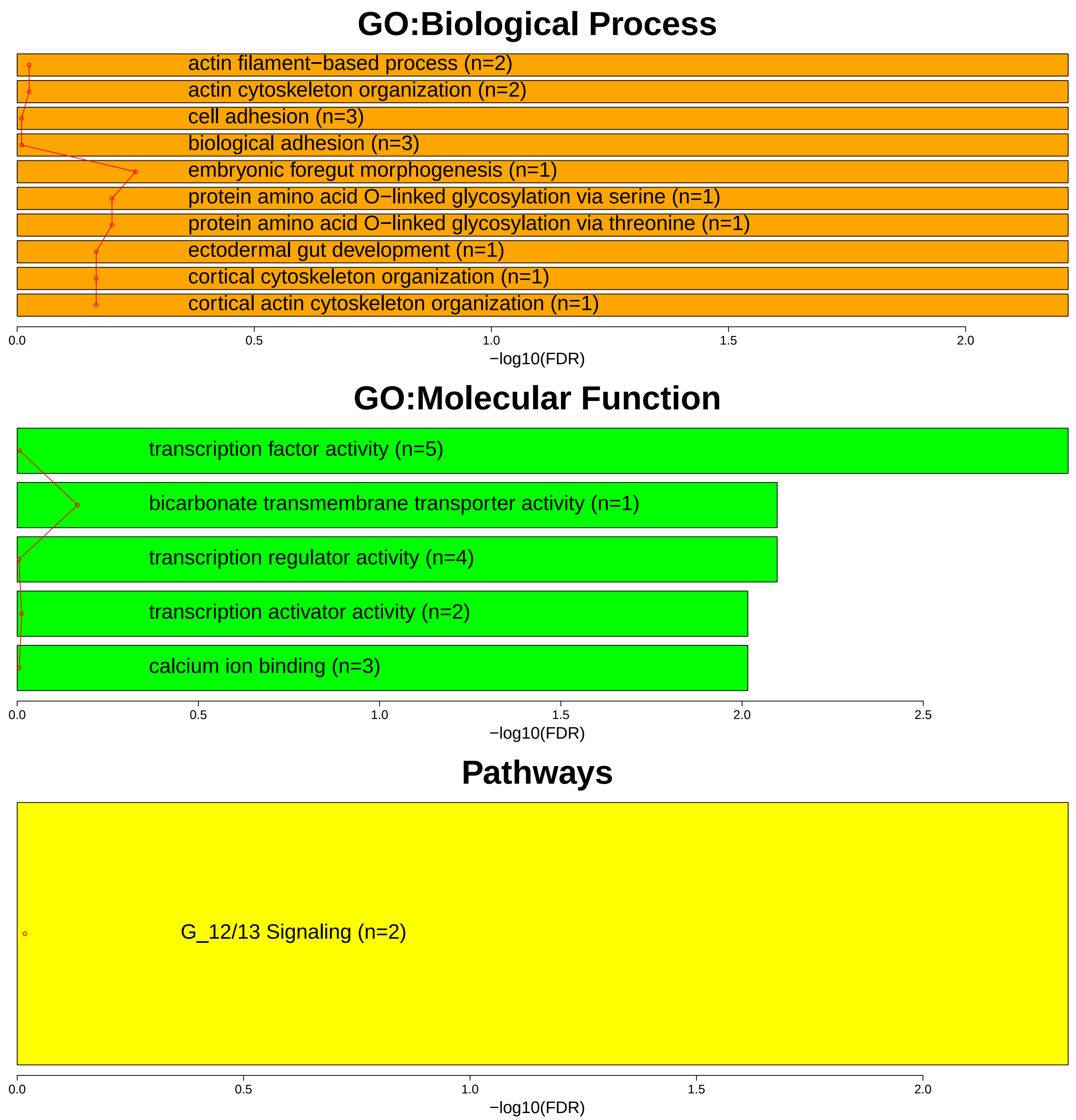

Figure S4: Enrichment analysis of GO terms and pathways for validated DMGs active in one of the subgroups of the TCGA-COAD samples.

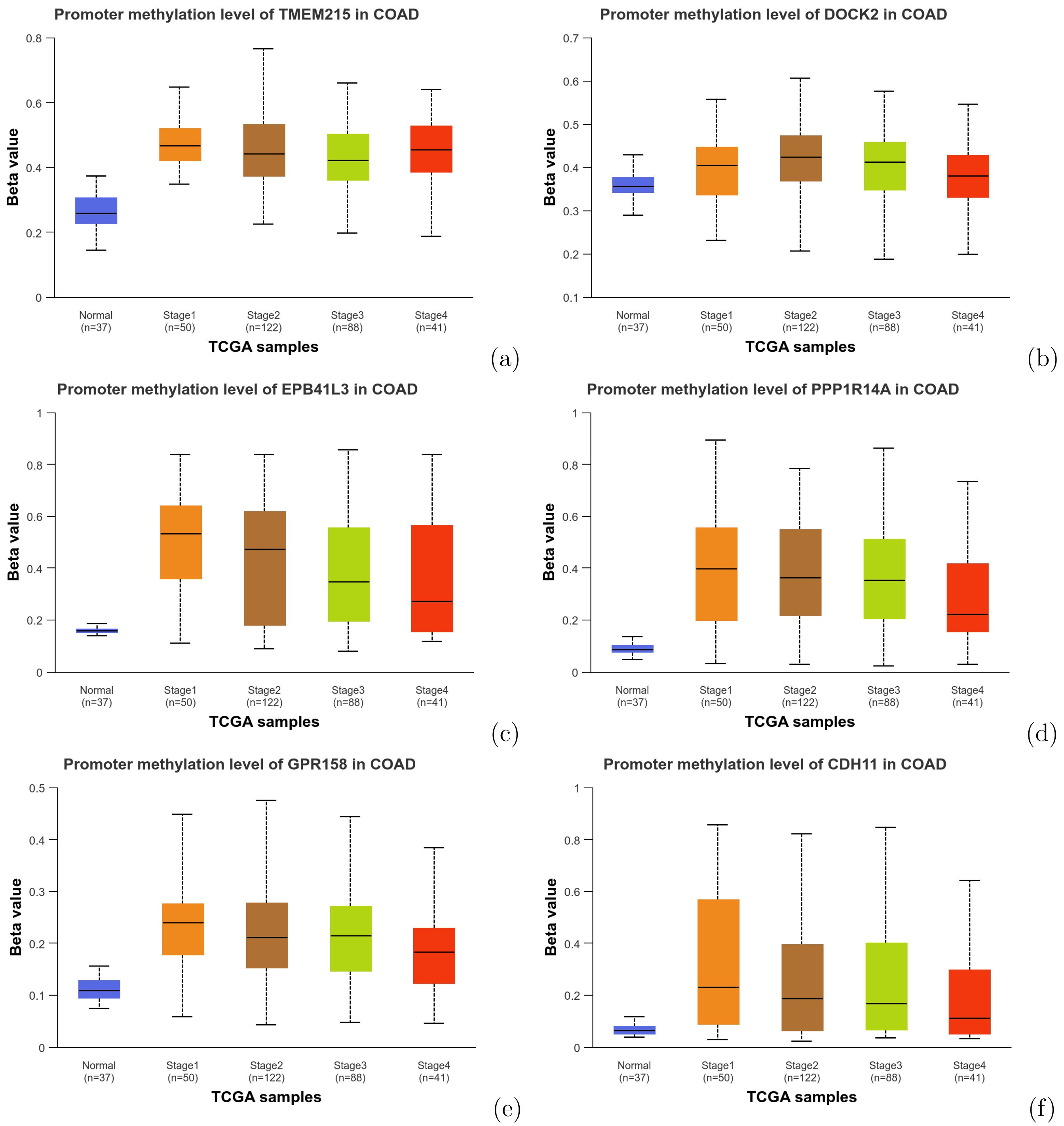

Figure S5: DNA methylation level of genes TMEM215(a), DOCK2(b), EPB41L3(c), PPP1R14A(d), GPR158(e) and CDH11(f) based on stages of CRC.

S2 Tables

Table S1: Percentage of CpGs identified as DMCs by `DMCTHM` in CRC separated by Chromosomes.

**Chr**	Percentage ($FDR < 0.05$)	Percentage ($FDR < 0.01$)	**Chr**	Percentage ($FDR < 0.05$)	Percentage ($FDR < 0.01$)	**Chr**	Percentage ($FDR < 0.05$)	Percentage ($FDR < 0.01$)
1	5.28	0.72	9	6.45	0.70	17	2.80	0.30
2	8.08	0.74	10	8.87	1.13	18	13.91	0.98
3	5.48	0.63	11	8.86	0.80	19	4.01	0.63
4	13.82	1.37	12	5.46	0.64	20	12.50	2.51
5	11.38	0.79	13	15.99	2.41	21	14.54	1.39
6	6.54	0.81	14	7.26	0.91	22	6.53	0.70
7	11.63	1.99	15	6.24	0.66	X	13.31	1.80
8	12.60	1.47	16	7.42	1.25	Y	24.25	12.45

Table S2: Percentage of CpGs identified as DMCs by `DMCTHM` in ACF separated by Chromosomes

**Chr**	Percentage ($FDR < 0.05$)	Percentage ($FDR < 0.01$)	**Chr**	Percentage ($FDR < 0.05$)	Percentage ($FDR < 0.01$)	**Chr**	Percentage ($FDR < 0.05$)	Percentage ($FDR < 0.01$)
1	0.34	0.17	9	0.40	0.19	17	0.46	0.19
2	0.43	0.21	10	0.76	0.37	18	0.85	0.52
3	0.30	0.17	11	0.46	0.22	19	0.55	0.27
4	0.60	0.30	12	0.48	0.25	20	0.66	0.33
5	0.54	0.25	13	0.80	0.36	21	0.64	0.44
6	0.64	0.36	14	0.45	0.23	22	0.42	0.19
7	0.42	0.19	15	0.47	0.24	X	12.0	0.06
8	0.71	0.38	16	0.39	0.16	Y	0.00	0.00

Table S3: DMC identification via `DMCTHM` vs T-test, classified by genomic location and methylation direction in the CRC dataset.

	`DMCTHM`	DMC						NDMC					
	T-test	DMC			NDMC			DMC			NDMC		
Type	Direction	Equal	Hyper	Hypo	Equal	Hyper	Hypo	Equal	Hyper	Hypo	Equal	Hyper	Hypo
All CpGs	Equal	0	0	0	0	0	0	0	89070	83959	19999661	0	0
	Hyper	0	35355	53	171193	0	0	0	0	0	0	0	0
	Hypo	0	125	27130	1643441	0	0	0	0	0	0	0	0
Islands	Equal	0	0	0	0	0	0	0	68983	7354	3255545	0	0
	Hyper	0	32695	42	140326	0	0	0	0	0	0	0	0
	Hypo	0	13	1661	44496	0	0	0	0	0	0	0	0
Promoter	Equal	0	0	0	0	0	0	0	24019	6620	2324726	0	0
	Hyper	0	12140	17	60138	0	0	0	0	0	0	0	0
	Hypo	0	8	1406	44969	0	0	0	0	0	0	0	0
Intergenic	Equal	0	0	0	0	0	0	0	63640	74376	17607538	0	0
	Hyper	0	23957	32	119597	0	0	0	0	0	0	0	0
	Hypo	0	118	24466	1588714	0	0	0	0	0	0	0	0
Intron	Equal	0	0	0	0	0	0	0	36816	32823	9670388	0	0
	Hyper	0	14837	22	67674	0	0	0	0	0	0	0	0
	Hypo	0	40	9692	536376	0	0	0	0	0	0	0	0
Exon	Equal	0	0	0	0	0	0	0	25503	9613	2401258	0	0
	Hyper	0	11429	21	51741	0	0	0	0	0	0	0	0
	Hypo	0	7	2679	55038	0	0	0	0	0	0	0	0
NShore	Equal	0	0	0	0	0	0	0	11650	7961	1301015	0	0
	Hyper	0	3713	6	25058	0	0	0	0	0	0	0	0
	Hypo	0	11	2156	40910	0	0	0	0	0	0	0	0
SShore	Equal	0	0	0	0	0	0	0	10845	8387	1312574	0	0
	Hyper	0	3723	6	24458	0	0	0	0	0	0	0	0
	Hypo	0	5	2165	41529	0	0	0	0	0	0	0	0
NShelf	Equal	0	0	0	0	0	0	0	9606	5503	969528	0	0
	Hyper	0	3068	4	17391	0	0	0	0	0	0	0	0
	Hypo	0	8	1716	34329	0	0	0	0	0	0	0	0
SShelf	Equal	0	0	0	0	0	0	0	9461	5941	970087	0	0
	Hyper	0	3600	6	17967	0	0	0	0	0	0	0	0
	Hypo	0	15	1735	33925	0	0	0	0	0	0	0	0
